# Suramin binds and inhibits infection of SARS-CoV-2 through both spike protein-heparan sulfate and ACE2 receptor interactions

**DOI:** 10.1038/s42003-023-04789-z

**Published:** 2023-04-08

**Authors:** Paul S. Kwon, Shirley Xu, Hanseul Oh, Seok-Joon Kwon, Andre L. Rodrigues, Maisha Feroz, Keith Fraser, Peng He, Fuming Zhang, Jung Joo Hong, Robert J. Linhardt, Jonathan S. Dordick

**Affiliations:** 1 Department of Chemistry and Chemical Biology, Rensselaer Polytechnic Institute, Troy, NY USA; 2 Department of Chemical and Biological Engineering, Center for Biotechnology and Interdisciplinary Studies, Rensselaer Polytechnic Institute, Troy, NY USA; 3 National Primate Research Center, Korea Research Institute of Bioscience and Biotechnology, Cheongju, Chungcheongbuk Republic of Korea; 4 College of Veterinary Medicine, Chungbuk National University, Cheongju, Chungcheongbuk Republic of Korea; 5 Department of Biological Sciences, Rensselaer Polytechnic Institute, Troy, NY USA; 6 Department of Biomedical Engineering, Rensselaer Polytechnic Institute, Troy, NY USA; 7 Present Address: Department of Biochemistry, University of Washington, Seattle, WA 98195 USA

**Keywords:** Target validation, Glycobiology

## Abstract

SARS-CoV-2 receptor binding domains (RBDs) interact with both the ACE2 receptor and heparan sulfate on the surface of host cells to enhance SARS-CoV-2 infection. We show that suramin, a polysulfated synthetic drug, binds to the ACE2 receptor and heparan sulfate binding sites on the RBDs of wild-type, Delta, and Omicron variants. Specifically, heparan sulfate and suramin had enhanced preferential binding for Omicron RBD, and suramin is most potent against the live SARS-CoV-2 Omicron variant (B.1.1.529) when compared to wild type and Delta (B.1.617.2) variants in vitro. These results suggest that inhibition of live virus infection occurs through dual SARS-CoV-2 targets of S-protein binding and previously reported RNA-dependent RNA polymerase inhibition and offers the possibility for this and other polysulfated molecules to be used as potential therapeutic and prophylactic options against COVID-19.

## Abstract (web summary)

Suramin, a polysulfated synthetic drug, binds to the ACE2 receptor and heparan sulfate binding sites on SARS-CoV-2 RBDs with preferential binding for Omicron RBD and inhibition of infection by the Omicron variant in vitro.

## Introduction

SARS-CoV-2 variants continue to emerge due to increased mutational pressures from susceptible populations^1^ and immune evasion^2^ making natural immunity^3^ and existing vaccines less effective^4^, resulting in breakthrough infections. For these reasons, broadly neutralizing therapeutics are of interest in combating existing and emerging SARS-CoV-2 variants. Protein-based approaches based on angiotensin‐converting enzyme 2 (ACE2) receptor mimicry include antibodies^5^, de novo minibinders^6^, and multivalent versions of designed minibinders^7^. Drug repositioning is another potential strategy for the discovery of non-protein, broad-spectrum therapeutic candidates. Yin et al.^8^ discovered that one such compound, suramin, inhibited RNA-dependent RNA polymerase (RdRp) by preventing binding of the RdRp with the RNA template, blocking viral replication. In addition, Salgado-Benvindo et al.^9^ demonstrated that suramin interferes with viral entry into Vero E6 and human airway epithelial cells. However, the specific nature of this interference remains unknown.

Our group^10^ and others^11^ have found that polysulfates and polysaccharides with *O*-linked sulfo groups, including heparin, pentosan and mucopolysaccharide polysulfate^12^, algal-derived fucoidans^13^ and rhamnan sulfate^14^, bind to the SARS-CoV-2 spike protein (S-protein) and inhibit viral entry through selective electrostatic interactions. Indeed, SARS-CoV-2 S-protein, through its receptor-binding domain (RBD), interacts with negatively charged cellular heparan sulfate (HS)^15,16^. SARS-CoV-2 virions enrich their local concentration through these non-specific interactions before binding to the ACE2 receptor. Docking studies suggest that heparin or HS bind to a site adjacent to the ACE2 binding site, and this is substantiated by experimental results showing ACE2 and HS can independently bind the S-protein^15,17^. In the present work, we examined the ability of suramin to bind to RBDs of several SARS-CoV-2 variants and elucidated the mechanism by which this potential repositioned drug may serve to inhibit viral entry and propagation.

## Results

### Suramin binds to SARS-CoV-2 spike protein to block virus-cell interactions

**Fig. 1:**
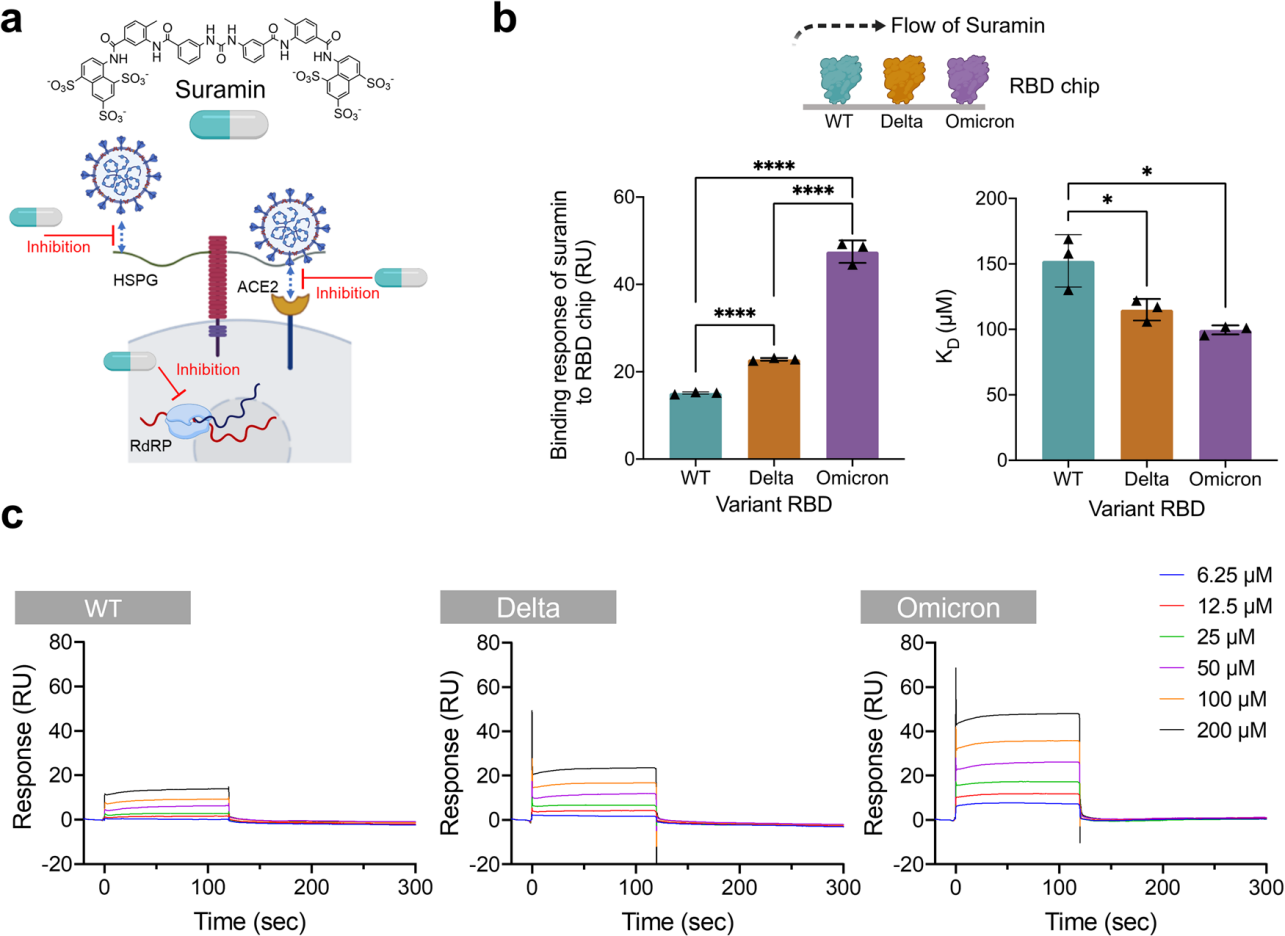
Suramin interacts with SARS-CoV-2 RBD variants for inhibiting SARS-CoV-2 cell entry. **a** Structure of suramin and proposed targets. Figure created with Chemdraw and Biorender.com. **b** SPR assay used to determine the binding affinity of suramin with immobilized SARS-CoV-2 variant RBDs (wild type (WT), Delta, and Omicron) on-chip (left panel). Equilibrium binding constants of suramin to RBD of different variants (right panel). **c** Sensorgrams for binding of suramin to SARS-CoV-2 variants. Various concentrations of suramin (6.25–200 µM in PBS) were flowed over an RBD chip containing WT, Delta, and Omicron variants on different flow cells. For these experiments *n* = 3 biological replicates. Statistical significance was determined using a student’s *t*-test with *p* > 0.05 = ns, *p < 0.05, **p < 0.01, ***p < 0.001, and ****p < 0.0001.

Suramin is a synthetic polysulfonate consisting of six *C*-linked sulfo groups (Fig. 1a) and is used to treat African sleeping sickness^18^ and river blindness^19^. It is listed on the World Health Organization’s list of essential medicines^20^. Suramin exhibits a large range of target promiscuity, inhibiting dengue, herpes simplex, hepatitis, chikungunya, Ebola, and Zika viruses^21^. Given its polyanionic character, we hypothesized that suramin could bind the SARS-CoV-2 S-protein at its HS binding site and block virus-cell interactions, thereby addressing one key mechanism for preventing viral replication (Fig. 1a). To test this hypothesis, we investigated the ability of suramin to bind the RBDs of wild type, Delta (B.1.617.2), and Omicron (B.1.1.529) SARS-CoV-2 variants (Supplementary Table 1). We used surface plasmon resonance (SPR) with RBDs of WT, Delta and Omicron S-protein immobilized on the SPR chip to assess binding of suramin. Binding affinity for the Omicron variant was approximately 50% stronger than for WT and over 30% stronger than the Delta variant (Fig. 1b), which is consistent with native-PAGE analysis of suramin-RBD variant complexes (Supplementary Fig. 1). In addition, binding affinity of suramin to RBD variants is not sensitive to the ionic strength up to 500 mM NaCl, with some reduction in binding affinity at 1 M NaCl (Supplementary Fig. 2). Moreover, suramin bound to the variants in a dose-dependent manner (Fig. 1c).

Along with the D614G mutation that confers higher viral transmissibility^22^, the SARS-CoV-2 Delta variant S-protein contains two positively charged RBD mutations, L452R and T478K (Supplementary Table 1)^23^. These additional positive charges in the Delta variant are likely responsible for tighter binding to the negatively charged suramin sulfo groups as compared with the WT. The S-protein has undergone even more extensive mutations in the Omicron variant^24^; 15 amino acid changes are located on the RBD alone, five of which (G339D, N440K, S477N, T478K and N501Y) are known to enhance ACE2 receptor binding^25^, and four mutations (N440K, T478K, Q493R, and Q498R) result in the S-protein gaining a substantial level of additional positive charges. This is consistent with the ability of suramin to bind to the Omicron RBD more tightly than to either Delta or WT.

**Fig. 2:**
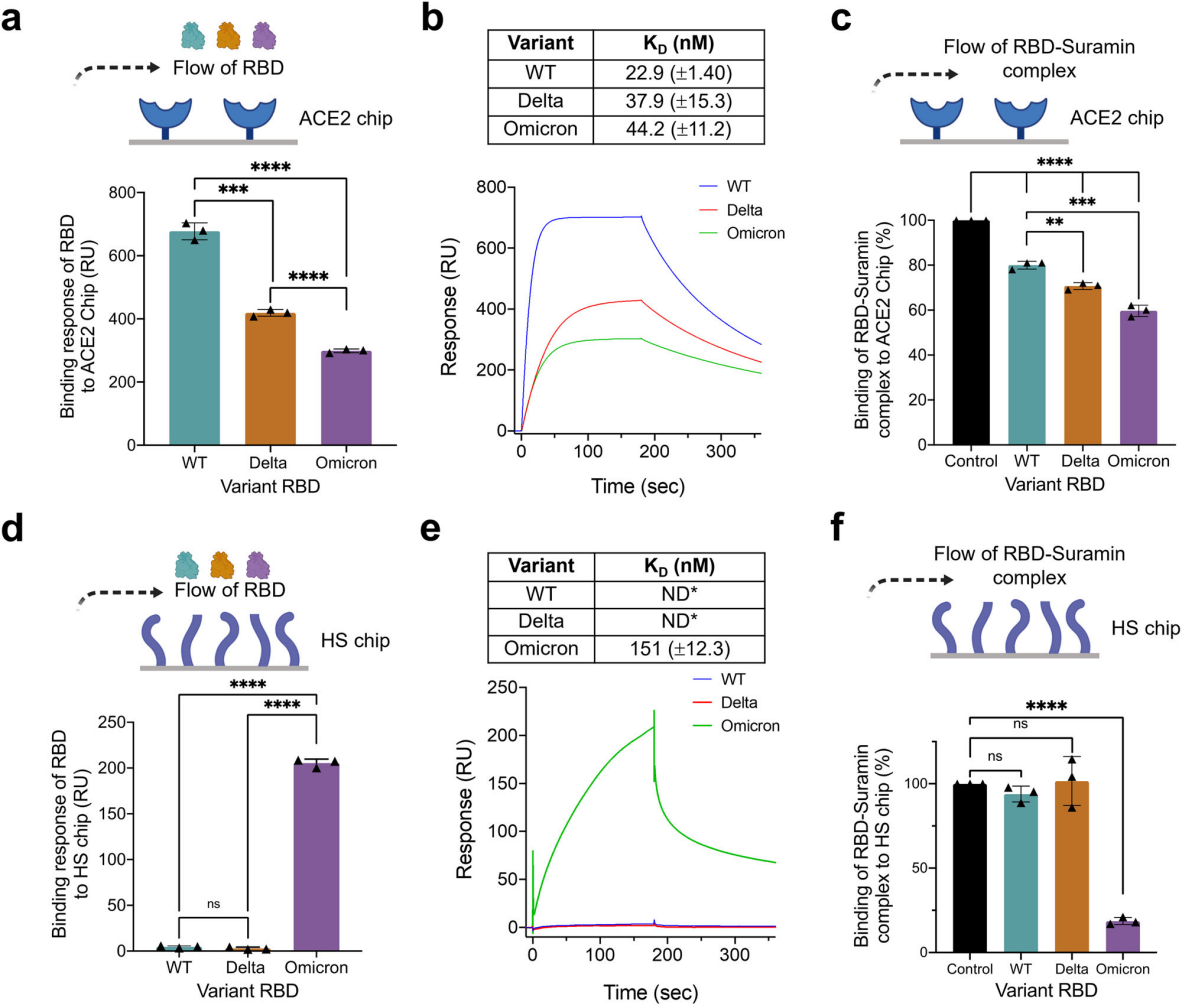
Suramin-SARS-CoV-2 RBD variant complexes inhibit the binding to both ACE2 and HS. **a** SPR assay used to determine the binding affinity of SARS-CoV-2 variant RBDs (wild type (WT), Delta, and Omicron) with immobilized ACE2 on-chip. **b** Binding response curves of different RBD variants to ACE2 chip. Resulting sensorgram of binding at 250 nM of RBD in HBS-EP + buffer (pH 7.4); K_D_ values determined through fitting to 1:1 Langmuir binding model are shown in the table. For these experiments *n* = 3 biological replicates. **c** SPR competition assay between suramin and immobilized ACE2 for SARS-CoV-2 variant RBD binding. **d** SPR assay used to determine the binding affinity of SARS-CoV-2 variant RBDs with immobilized heparan sulfate (HS) on-chip. **e** Binding response curves of different RBD variants to HS chip. Resulting sensorgram of binding at 250 nM of RBD in HBS-EP + buffer (pH 7.4); K_D_ values determined through fitting to 1:1 Langmuir binding model are shown in the table. Note that for WT and Delta, K_D_ was undetermined in the range of RBD concentrations as binding to HS was too low (ND* indicates “not determined”). For these experiments *n* = 3 biological replicates. **f** SPR competition assay between suramin and immobilized HS for SARS-CoV-2 variant RBD binding. Data are presented as mean ± s.d., *n* = 3 biologically independent samples. Statistical significance was determined using a student’s *t*-test with *p* > 0.05 = ns, *p < 0.05, **p < 0.01, ***p < 0.001, and ****p < 0.0001. Error bars show mean ± sd.

SPR was then used to probe RBD binding to ACE2. The tightest interaction was observed with WT RBD (Fig. 2a, b). As the number of mutations increased from Delta to Omicron, the RBD binding affinity with ACE2 decreased. To assess whether suramin interacts at the ACE2 binding site, we performed competition SPR experiments with pre-incubated suramin-RBD complexes and immobilized ACE2. ACE2 was able to displace suramin in all three variant RBDs and suramin was most effective in reducing binding to ACE2 against the Omicron variant. (Fig. 2c and Supplementary Fig. 3). Competitive inhibition by suramin of wild-type RBD binding to ACE2 was also observed in the presence of 0.5% (w/v) mucin, the major protein component of mucus (Supplementary Fig. 4). We next investigated whether the RBDs could bind immobilized HS (Fig. 2d, e). Astonishingly, very weak HS binding was observed with WT and Delta RBDs, while Omicron RBD bound very tightly to HS at the concentrations tested (Fig. 2e). HS as an enhanced co-receptor for Omicron may account for differences in transmissibility even with reduced ACE2 affinity^26^ (Fig. 2) when compared to WT and Delta. Finally, as in the ACE2 competition assay, suramin was most effective against Omicron RBD in reducing HS binding (Fig. 2f and Supplementary Fig. 5).

### Molecular docking of suramin to S-protein RBD

**Fig. 3:**
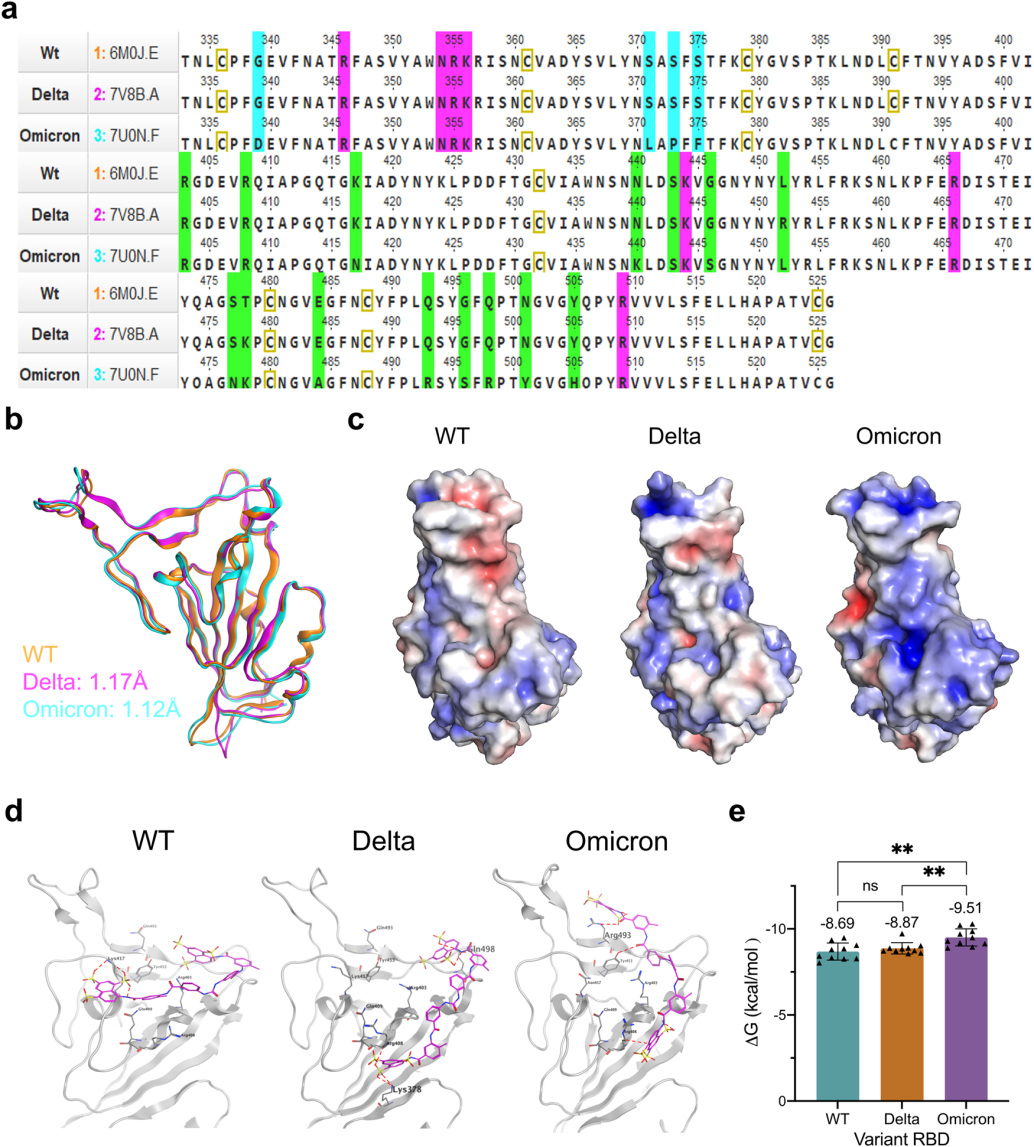
Structural and molecular docking analysis of suramin and SARS-CoV-2 RBD variant complexes. **a** Sequence alignment of wild type (WT) (6M0J), Delta (7V8B) and Omicron (7U0N) RBDs. **b** Superimposed structures of WT, Delta, and Omicron RBDs optimized by using the mean square distance (MSD) deviation of corresponding C-α carbons. **c** Electrostatic maps for variant RBDs facing ACE2 binding site. Electrostatic surface potentials are colored red and blue for negative and positive charges, respectively, and white represents neutral residues. **d** Binding of suramin in complex with the S-protein RBDs of wild-type (PDB: 6M0J), Delta (PDB: 7V8B), and Omicron (PDB: 7U0N) SARS-CoV-2 variants. **e** Global free energy was determined by docking modeling of suramin binding to the ACE2 binding site of each RBD. Error bars show mean ± sd.

To probe the impact of suramin on the RBD-ACE2 interaction, we performed molecular docking after structural alignment of the RBD region of the S-protein from the wild-type (PDB ID: 6M0J)^27^, Delta B.1.617.2 (PDB ID: 7V8B) and Omicron BA.1 (PDB ID: 7U0N)^28^ variants, which revealed high structural homology (RMSD < 0.9) (Fig. 3a, b). On each variant RBD we observed tighter binding of suramin to the ACE2 binding site as a function of increased positive charge density from wild-type to Delta to Omicron (Fig. 3). The positively charged electrostatic surfaces (Fig. 3c) of Delta (L452R and T478K) and Omicron RBD (N440K, T478K, Q493R, Q498R, and Y505H) allow for a change in suramin binding conformation, resulting in tighter binding. In particular, the Arg493 residue in Omicron RBD directly interacts with one of the sulfate groups of suramin (Fig. 3d). We also observed this in molecular docking of suramin to ACE2, which gives a more negative ΔG for Omicron (−9.51 ± 0.50 kcal/mol) compared to that of wild-type (−8.69 ± 0.50 kcal/mol) and Delta (−8.87 ± 0.33 kcal/mol) (Fig. 3e). The observed disparity in the binding of suramin to each RBD suggests that suramin is able to bind and act as an “electrostatic shield” to prevent subsequent binding of HS. SPR experiments and docking studies suggest that entry inhibition occurs by blocking both ACE2 and HS binding sites with more efficient inhibition observed on the Omicron variant.

### Suramin as an anti-SARS-CoV-2 agent

**Fig. 4:**
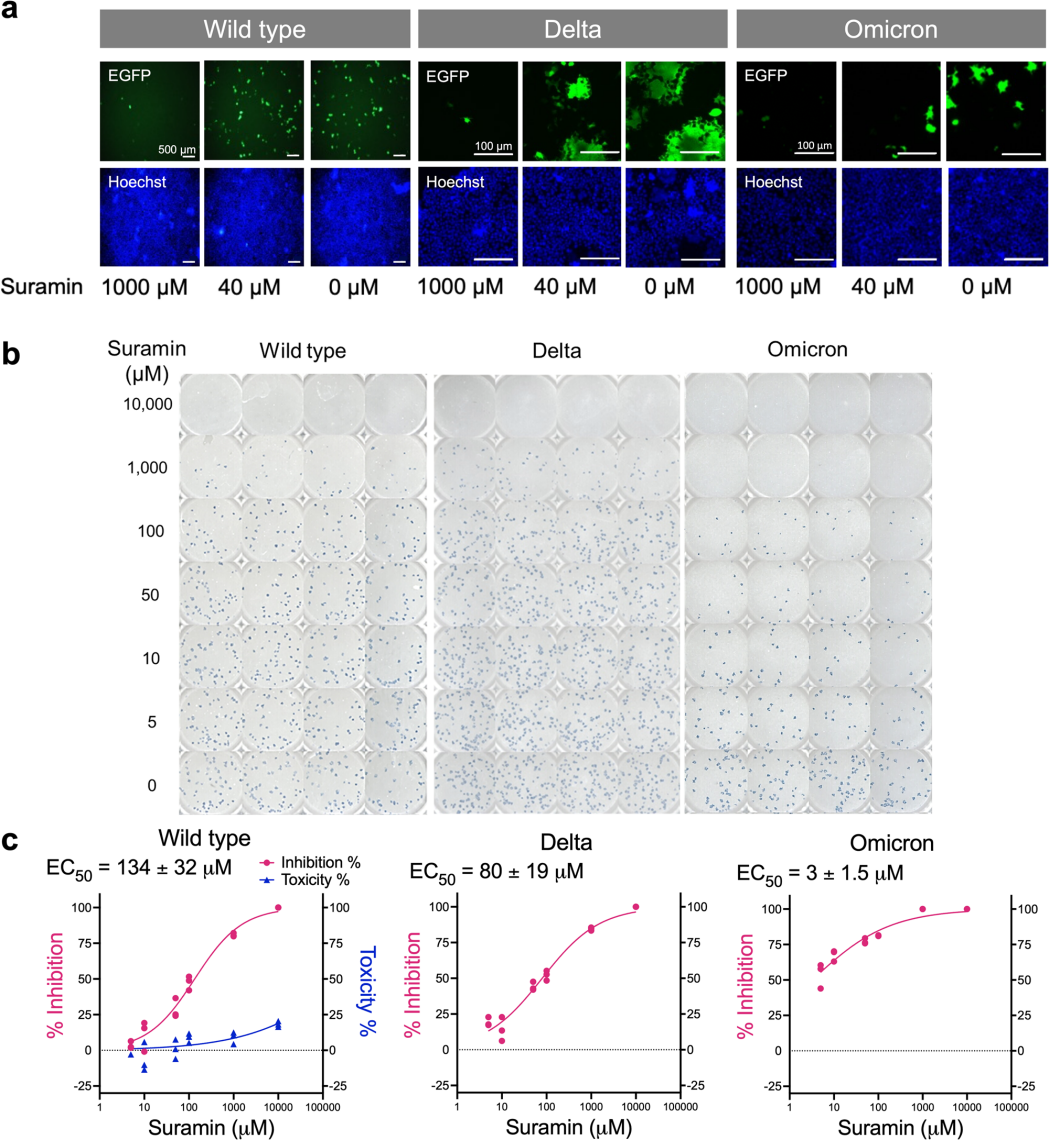
Assessment of antiviral activities of suramin. **a** In vitro SARS-CoV-2 pseudotyped viral particle neutralization assays against wild type (WT), Delta, and Omicron variants. Representative fluorescence microscopy images as a function of suramin concentration. **b** Focus reduction assay images of the infection by live SARS-CoV-2 variants upon treatment of suramin. At 2-days after infection, Vero cells were fixed and probed with SARS-CoV-2 spike primary antibody (1:10000, Sino Bio Inc.) and HRP-conjugated goat rabbit (1:10000, Abcam) secondary antibody. **c** Vero cells were infected with SARS-CoV-2 at a 50 focus-forming unit (FFU) of wild type at different doses of suramin for 48 h. The viral yield was quantified using a focus reduction assay. Cytotoxicity in Vero cells was measured using the WST-1 assay. The left and right y-axes represent mean percent inhibition of virus yield and cytotoxicity of the polysaccharides, respectively.

Standard assays were performed using pseudotyped and live virus particles to determine whether the in vitro binding studies on suramin translated to antiviral activity. Pseudotyped viral particles provide a unique opportunity to evaluate SARS-CoV-2 cellular infection as a result of the S-protein alone by uncoupling viral entry and RdRp inhibitory mechanisms. Pseudotyped viral particles encapsulating an *Egfp* reporter gene were used to infect HEK293T cells expressing ACE2 after pre-incubation with varying concentrations of suramin (Fig. 4a). Suramin exhibited broad entry inhibition among all three variants tested.

Based on the pseudotyped virus results, we tested suramin against the live SARS-CoV-2 virus using a focus reduction assay on Vero E6 cells (Fig. 4b). This assay revealed dose-dependent suramin antiviral activity in WT with an EC_50_ of 134 ± 32 µM, Delta with an EC_50_ of 80 ± 19 µM, and Omicron with an EC_50_ of 3.0 ± 1.5 µM (Fig. 4c). These results correlated with the binding studies (Fig. 2c, f) and showed that suramin is a potent SARS-CoV-2 infection inhibitor across variants of SARS-CoV-2 and is most active against the Omicron variant.

In addition to virus neutralization, dose-dependent cytotoxicity was examined in Vero E6 cells using the standard tetrazolium salt-1 (WST-1) assay and indicated very low cytotoxicity (<20%) even at suramin concentrations up to 10 mM (Fig. 4c). RdRp sequences are highly conserved among the variants tested (Supplementary Fig. 6a). The G671S mutation found in the delta strain and the P323L mutation found in the delta and omicron strains are distal to the suramin binding sites on the RdRp (Supplementary Fig. 6b). As a result, we hypothesized that the observed differences in efficacy among the SARS-CoV-2 variants tested predominantly occur through suramin-RBD interactions.

## Discussion

In this study, we show that the synthetic polyanionic drug suramin effectively binds to the RBD of the SARS-CoV-2 S-protein, suggesting a second mechanism for suramin inhibition of viral replication. The binding affinity of suramin to the RBD of S-protein variants was strongest for the Omicron variant, intermediate for the Delta variant, and weakest for WT, which tracks with the degree of infectivity among the tested variants. S-protein-suramin complexes competitively inhibited the binding of ACE2 even in the presence of mucin or high ionic strength. Suramin broadly inhibited the infection of live SARS-CoV-2 in increasing order of potency for Omicron > Delta > WT. SPR experiments and docking studies suggest that entry inhibition occurs by blocking both ACE2 and HS binding sites in the Omicron variant while Delta and WT predominantly block ACE2 interactions alone.

The ongoing emergence of new SARS-CoV-2 variants with mutations in the S-protein gene, which have become particularly effective in evading immunity from vaccination or prior infection remains a public health challenge. This complication further necessitates the identification of effective potential therapeutic interventions that are effective across emerging variants. Interestingly, as the virus has evolved, it appears that the RBD has become more positively charged, particularly in the region of its heparan sulfate binding sites. The enhanced basicity of key regions of the S-protein suggests that polyanionic molecules, such as suramin, may be particularly effective against existing and future SARS-CoV-2 variants. Since suramin is not approved in the United States due to toxicity concerns^29^, further pharmacological studies and clinical trials are needed to demonstrate the effectiveness of repurposed suramin as a COVID-19 therapeutic or as a post-exposure prophylaxis, for example as a nasal spray.

## Materials and methods

### Suramin binding to SARS-CoV-2 RBD Variants via SPR

RBDs for wild type, Delta, and Omicron variants of SARS-Cov-2 were expressed in Expi293F cells and purified in the Bates lab (University of Mississippi Medical Center). CM5 sensor chips and EDC/NHS for amine coupling were purchased from Cytiva (Marlborough, MA, USA). To prepare RBD chips, each variant RBD was diluted to a concentration of 200 µg/mL using sterilized PBS (pH 7.4). RBDs were immobilized onto the chip using amine coupling as described by the manufacturer’s protocol using the Biacore T200 machine (Cytiva, Marlborough, MA, USA). A reference channel was prepared by activating and deactivating the chip surface using the same amine coupling method. Suramin was prepared as a stock solution of 20 mM in DI water and diluted to different concentrations with running buffer. Using the immobilized chip, suramin samples of different concentration (0–200 µM) was injected onto the chip at a flow rate of 30 µL/min for a 2 min association phase. Afterward, running buffer was used to dissociate suramin on the chip for a 3 min dissociation phase. After each association–dissociation cycle, the surface of the chip was regenerated using 30 µL of 2 M NaCl. All experiments were performed at 25 °C using the Biacore T200 machine. The resulting sensorgrams were analyzed using the Biacore T200 evaluation software. All sensorgrams were reference subtracted using the reference channel and blank subtracted using the resulting sensorgram for the buffer cycle (no suramin). Sensorgrams showed rapid saturation, so K_D_ was determined by fitting sensorgrams to a steady-state kinetic model.

### Native PAGE and western blot of suramin-RBD interaction

Wild-type, Delta, or Omicron RBD (each at 500 nM) was prepared by diluting in PBS (pH 7.4). Suramin was prepared by dissolving up to 2 µM using PBS. RBD and suramin were mixed for a final concentration of 250 mM RBD and 1 µM suramin and incubated at room temperature for 30 min. After incubation, a native gel was performed, and the gel was transferred onto a nitrocellulose membrane using the BioRad Trans-Blot Turbo Transfer System. After transfer, the blot was blocked with 5% skim milk in PBS with 0.2% Tween-20 (PBST, pH 7.4) for 1 h at room temperature under gentle rocking conditions. Afterwards, anti-His primary antibody (1:2000 dilution factor, SAB1305538, Sigma, St. Louis, MO) in 1% skim milk in PBST was added to the blot and incubated overnight at 4 °C under gentle rocking conditions. After overnight incubation, the blot was washed with five 10 min washes using PBST. Goat anti-mouse HRP conjugated secondary antibody (1:10,000 dilution factor, 31430, Thermo Fisher Scientific, Waltham, MA) in 1% skim milk in PBST was added to the blot and incubated for 1 h at room temperature under gentle rocking conditions. Prior to adding substrate, the blot was washed again with five 10 min washes using PBST. SuperSignal™ West Pico PLUS Chemiluminescent substrate (Thermo Fisher Scientific) was added to the blot and was imaged using the BioRad Gel Doc XR + System (BioRad, Hercules, CA).

### Effect of salt concentrations on binding of suramin to RBD

HBS-EP + buffer with different salt concentrations (150 mM, 300 mM, 500 mM, and 1000 mM) was prepared. Suramin (0 – 200 µM) was prepared by diluting the stock solution into the different HBS-EP + buffers. Suramin was injected over the RBD chip at 30 µL/min for a 3 min association phase followed by a 3 min dissociation phase using running buffer (HBS-EP + ). Regeneration of the chip was performed by injecting 2 M NaCl for 1 min at the same flow rate. All experiments were performed at 25 °C using a Biacore T200 machine. The resulting sensorgrams were analyzed using the Biacore T200 evaluation software. K_D_ was determined by fitting sensorgrams to a steady-state kinetic model.

### Preparation of heparan sulfate (HS) and ACE 2 SPR chips

Biotinylated HS was prepared by reacting amine-PEG3-biotin (Thermo Fisher Scientific, Waltham, MA, USA) with the reducing end of HS (Celsus laboratories, Inc., Cincinnati, OH, USA) through reductive amination. The procedure was as follows: 1 mg of amine-PEG3-biotin and 5 mg of NaBH_3_CN were added to the stirred HS solution (1 mg HS in 0.4 mL water). Then, the reaction was incubated at 70 ^o^C for 24 h, after which an additional 5 mg NaBH_3_CN (in 0.2 mL water) was added to continue the reaction for another 24 h. The resulting solution was desalted by Microsep 1 kDa MWCO yellow Omega (polyethersulfone-based) centrifugal device (Pall Filtron Corporation, Port Washington, NY, USA). To prepare a HS chip, the solution of biotinylated HS (1 mg/mL) in running buffer, HBS-EP + (0.01 M 4-(2-hydroxyethyl)-1-piperazineethanesulfonic acid, 0.15 M NaCl, 3 mM ethylenediaminetetraacetic acid, 0.05% surfactant P20, pH 7.4), was injected over flow cells 2, 3, 4 (FC2, FC3, FC4) of a SA chip (Cytiva) for 2 min at a flow rate of 10 µL/min. The successful immobilization of HS was confirmed by the observation of a ~150-200 resonance unit (RU) increase in the sensor chip. Remaining streptavidin on FC2-4 was blocked with saturated biotin (in HBS-EP + ) for 1 min at the same flow rate. Reference channel (FC1) was prepared using 2 min injection of saturated biotin at the same flow rate.

For the ACE2 SPR chip, biotinylated ACE2 was purchased from Sino Biologicals (Beijing, China). ACE2 chip preparation was performed as indicated for the HS chip. Briefly, biotinylated ACE2 was injected over flow cells 2-4 (FC 2-4) at a flow rate of 10 µL/min for 2 min. A RU increase of 2000 RU was observed indicated successful immobilization. Saturated biotin was injected over the same flow cells for 1 min to block the remaining streptavidin. The reference channel was prepared using 2 min injection of saturated biotin at the same flow rate.

### Binding of SARS-CoV-2 RBD variants to ACE2 or HS using SPR analysis

Different concentrations of RBD (250–1000 nM), from the Bates Lab, was diluted using HBS-EP + buffer and injected over the sensor chip at a flow rate of 30 µL/min for 3 min as an association phase. Afterward, running buffer was used to dissociate RBD on the chip for a 3 min dissociation phase. Regeneration was performed using 2 M NaCl at the same flow rate for 1 min. All experiments were performed with the Biacore T200 at 25 °C. Resulting sensorgrams were analyzed using Biacore T200 Evaluation Software and kinetic parameters were determined by fitting to a 1:1 Langmuir binding model.

### Solution competition SPR analysis on the inhibition of suramin on RBD

RBD (250 nM) was pre-mixed with various concentrations of suramin in HBS-EP + buffer. The mixture was injected over either ACE2 or HS chip at a flow rate of 30 µL/min for 3 min at 25 °C followed by a 3 min dissociation phase. The chip was regenerated after each cycle using 2 M NaCl for 1 min. To test the effect of mucin on binding, suramin (10 µM) was pre-mixed with RBD (250 nM) in HBS-EP + buffer containing 0.5% mucin. Similarly, the mixture was injected over an ACE2 chip at a flow rate of 30 µL/min for 3 min association and 3 min dissociation. Again, the chip was regenerated using 2 M NaCl for 1 min at the same flow rate. All experiments were performed with the Biacore T200 at 25 °C.

### Generation of HEK293T-ACE2 stable cell line

Lentiviral particles containing the ACE2-Puro construct were produced by transfecting 12.3 μg psPAX2 (Addgene# 12260), 2.5 μg pMD2g (Addgene# 12259), and 14.7 μg pLenti-hACE2-Puro (Addgene# 155295) into HEK293T cells using Lipofectamine 2000 according to manufacturer instructions. The harvest supernatant from HEK293T cells carrying the lentiviral particles was harvested at 48 h and 72 h. The supernatant was pooled together and concentrated using Lenti-X-Concentrator (Takara Bio, Kusatsu, Shiga, Japan) according to the manufacturer’s instructions. The concentrated lentiviral particles carrying ACE2-Puro were delivered to HEK293T cells on 6-well tissue culture-treated plates. After 48 h, 4 μg/mL of puromycin was added to DMEM + 10% FBS and a medium exchange was carried out. The cells were passaged into a T-25 flask and maintained in selection pressure (4 μg/mL puromycin) to remove cells lacking the ACE2-Puro construct.

### Construction of SARS-CoV-2 pseudovirus

To construct SARS-CoV-2 Pseudovirus bearing S protein (wild type, delta, and omicron), HEK293T cells were transfected with 26 µg of psPAX2, 26 µg of pLV-EGFP (a gift from Pantelis Tsoulfas, Addgene plasmid # 36083) and 8.7 µg of pHDM-SARS-COV2-S (BEI Resources #NR52514, wild type S-protein) or 8.7 µg of pHDM-Spike (delta and omicron) subcloned with the genes encoding S-protein variants (delta and omicron) synthesized from Twist Bioscience (South San Francisco, CA, USA). Lipofectamine 2000 (ThermoFisher Scientific) was used as the transfection reagent according to manufacturer’s protocol. A medium exchange was performed after 24 h with the addition of 5 mM sodium butyrate. The harvest supernatant was collected at 48 h and 72 h and pooled together and concentrated using the Lenti-X-Concentrator (Takara Bio, Shiga, Japan) according to manufacturer’s protocol. For titration of virus samples, HEK293T-ACE2 were seeded on tissue culture treated 96-well plates. The viral samples were introduced at three different dilutions (1:10, 1:50, and 1:100) to the cells. The cells were cultured for a period of 48 h and were then stained with 5 μg/mL Hoechst 33342 (Thermo Fisher Scientific, Waltham, MA, USA) and imaged using Cellomics Arrayscan XTI (Thermo Fisher Scientific, Waltham, MA, USA). The infection efficiency was then calculated using the Target Activation Bioapplication.

### Virus neutralization assay

Viral samples at 1:10 dilution were mixed with 5 different concentrations of suramin, that between 0.02–5 mM in a 5-fold serial dilution. Samples were diluted in serum-free Dulbecco’s Modified Eagle Medium (DMEM) (Thermo Fisher Scientific, Waltham, MA, USA). The mixture was then incubated at 37 °C for 1 h. The samples were then added to a HEK293T-ACE2 stable cell line previously established in our lab^30^. Cells were plated in 96-well plates at 5000 cells/well and incubated with suramin and virus solution for 4 h at 37 °C. Afterwards, media was changed for in DMEM + 10% FBS + 1% PenStrep and cultured for an additional 48 h. After 2 days of incubation, cells were then stained with 5 μg/mL of Hoechst 33342 and imaged using the Cellomics ArrayScan XTI. The infection efficiency was then calculated using the Target Activation Bioapplication. Percentage (%) of infected cells at each concentration was normalized to a negative control (no suramin). This control corresponded to the maximum fluorescence obtained in the assay. GraphPad Prism 8.0 (GraphPad Software) was used to fit sigmoidal dose-response curves to the average % inhibition and associated standard error of mean (SEM) to calculate EC_50_ values.

### Virus and propagation

Vero cells (Korean Cell Line Bank, KCLB No. 10081, Korea) were cultured in Dulbecco’s modified Eagle’s medium (DMEM), supplemented with 10% FBS, 50 U/ml penicillin, and 50 μg/ml streptomycin. SARS-CoV-2 isolates (NCCP no. 43326 for the S clade, NCCP no. 43390 for the GK clade [B.1.617.2 lineage, Delta], and NCCP no. 43408 for the GRA clade [B.1.1.529, Omicron]) were provided by the National Culture Collection for Pathogens. The wild-type virus or variants were propagated in Vero cells with DMEM + 2% FBS. At 72 h after infection, culture supernatants were collected and stored at −80 °C. Viral titer was determined by focus formation assay as described previously with slight modifications^10^. Ten-fold serial dilutions of wild-type virus or variants were incubated with the Vero cells for 1 h before further overlaying of 2% methyl cellulose in 4% FBS in DMEM. Subsequent procedures were conducted under the identical condition as described in Section of Focus reduction assay. Virus titers were calculated as focus forming units (FFU) per ml. All experiments with SARS-CoV-2 were conducted in a Biosafety Level 3 laboratory.

### Cytotoxicity assay

The cytotoxicity of suramin sodium salt on Vero cells was measured using the water soluble tetrazolium salt-1 (WST-1) assay (Takara Bio Inc., Kusatsu, Shiga, Japan) following the manufacturer’s protocol. Briefly, Vero cells were treated with several dose of each drug (5, 10, 50, 10^2^, 10^3^, 10^4 ^µM) for 48 h, and then incubated with premix WST-1 for 1 h at 37 °C. Absorbance was measured at 430 nm using ELISA plate reader (Epoch, Bio-Tek Instruments, Inc., Winooski, VT, USA). The 50% cytotoxic concentration for inhibitors (CC_50_; drug concentration that reduced the cell viability by 50% compared to the cell only control) was determined using nonlinear regression analysis (GraphPad Prism 8.4). Experiments were performed in duplicate wells and repeated three times.

### Focus reduction assay

Antiviral activity of suramin sodium salt was measured using focus reduction assay as described previously with some modifications. Briefly, different dose of the drug (5, 10, 50, 10^2^, 10^3^, 10^4 ^µM) containing about 50 FFU of wild-type virus or variants were pre-incubated for 1 h at 37 °C. Then, each mixture of virus and drug was added to each well and incubated for 1 h before further overlaying of 2% methyl cellulose in 4% FBS in DMEM. The plates were cultured at 37 °C for 2 days and then washed with cold PBS. The infected cells were fixed with 4% paraformaldehyde phosphate buffer solution for 30 min at 4 °C, followed by permeabilization with 0.5% Triton X-100 for 20 min at room temperature. After washing, SARS-CoV-2 Spike antibody (1:5000, Sino Bio Inc., Beijing, China) was added to each well and incubated for 45 min at room temperature. After repeating the washing, HRP-conjugated goat rabbit (1:10000, Abcam, Cambridge, UK) was added to each well for 45 min at room temperature. The foci were visualized by True Blue peroxidase substrate (KPL) for 20 min in the dark, and plate images were captured using an Immunospot CTL reader (S6 Universal analyzer) and the number of foci/well counted. SARS-CoV-2 was tested against each drug concentration in quadruplicate wells. The 50% inhibitory concentration of each drug against SARS-CoV-2 (IC_50_; drug concentration that inhibited FFU formation by 50% compared to the cell only control) was determined using non-linear regression analysis (GraphPad Prism 8.4).

### Structural analysis and molecular docking

The structures of the S-protein RBD from wild type (PDB ID: 6M0J) and Delta (B.1.617.2, PDB ID: 7V8B) variants were obtained from the protein data bank. Mutations observed in the RBD of the Omicron (B.1.1.529) S-protein were introduced onto the Omicron BA.1 structure (PDB ID: 7U0N) in Molecular Operating Environment (MOE) 2022 (Chemical Computing Group ULC, Montreal, QC, Canada) and a round of energy minimization was performed to generate a structure corresponding to the Omicron B.1.1.529 sequence used in this study. A blosum62 substitution matrix along with default alignment parameters was used to compare the sequences of the WT, Delta and Omicron RBDs. In addition to the MSA we superimposed the aligned structures of the three RBDs to determine the impact of the sequence mutations on the RBD structures. The mean squared distance (MSD) deviation between corresponding Cα carbons in each RBD was measured to enable a comparative analysis. Structures that are closely related would be expected to be separated by a distance of <2 Å. To further demonstrate the impact of the mutations across each strain we generated electrostatic maps of the RBDs using the Adaptive Poisson-Boltzman Solver (APBS) plugin in PyMol. The binding of suramin and heparan sulfate to each of the three RBDs was studied using the docking function in MOE2022.

### Statistics and reproducibility

Statistical analysis was performed using GraphPad Prism software. Other data were statistically analyzed by two-sided student’s *t*-test and were presented as mean ± SD. *P* < 0.05 was considered as statistical significance. *P < 0.05; **P < 0.01; ***P < 0.001; and ****P < 0.0001. Sample and replicate sizes were indicated in the figure legends.

### Reporting summary

Further information on research design is available in the Nature Portfolio Reporting Summary linked to this article.

## Supplementary information

Supplementary Material

Description of Additional Supplementary Files

Supplementary Data 1

Reporting Summary

## Data Availability

The datasets generated during and that support the findings of this study are available upon request. Source data for figures can be found in Supplementary Data.
