# Supplementary material for: Suramin binds and inhibits infection of SARS-CoV-2 through both spike protein-heparan sulfate and ACE2 receptor interactions
Source: Commun Biol. 2023 Apr 8;6:387. doi: 10.1038/s42003-023-04789-z (PMC10082822; doi:10.1038/s42003-023-04789-z)
Supplement: Supplementary file 1 — Supplementary Material [file 42003_2023_4789_MOESM1_ESM.pdf]

Supplementary Materials for:

Suramin binds and inhibits infection of SARS-CoV-2 through both spike protein-heparan sulfate and ACE2 receptor interactions

Paul S. Kwon[a,¶,^], Shirley Xu[b,¶], Hanseul Oh[c,d ¶], Seok-Joon Kwon[b,¶], Andre L Rodrigues[b], Maisha Feroz[b], Keith Fraser[e], Peng He[a], Fuming Zhang[b], Jung Joo Hong [d,*], Robert J. Linhardt[a,b,e,*], and Jonathan S. Dordick [b,e,f,*]

[a]Department of Chemistry and Chemical Biology, Rensselaer Polytechnic Institute, Troy, NY, USA

[b]Department of Chemical and Biological Engineering, Center for Biotechnology and Interdisciplinary Studies, Rensselaer Polytechnic Institute, Troy, NY, USA.

[c]National Primate Research Center, Korea Research Institute of Bioscience and Biotechnology, Cheongju, Chungcheongbuk, Republic of Korea.

[d]College of Veterinary Medicine, Chungbuk National University, Cheongju, Chungcheongbuk, Republic of Korea.

[e]Department of Biological Sciences, Rensselaer Polytechnic Institute, Troy, NY, USA

[f]Department of Biomedical Engineering, Rensselaer Polytechnic Institute, Troy, NY, USA

Supplementary Table 1. SARS-CoV-2 S-protein variants.

Variants	NTD (13-304)	RBD (319-540)	SD (541-683)	S2 subunit (685-1213)
Delta (B.1.617.2)	T19R, E156G, ∆157-158	L452R, T478K	D614G, P681R	D950N
Omicron (B.1.1.529)	67V, Δ69-70, T95I, G142D, Δ143-145, Δ211, L212I, ins214EPE	G339D, S371L, S373P, S375F, K417N, N440K, G446S, S477N, T478K, E484A, Q493R, G496S, Q498R, N501Y, Y505H	T547K, D614G, H655Y, N679K, P681H	N764K, D796Y, N856K, Q954H, N969K, L981F

NTD, N-terminal domain; RBD, receptor-binding domain; SD, subdomain

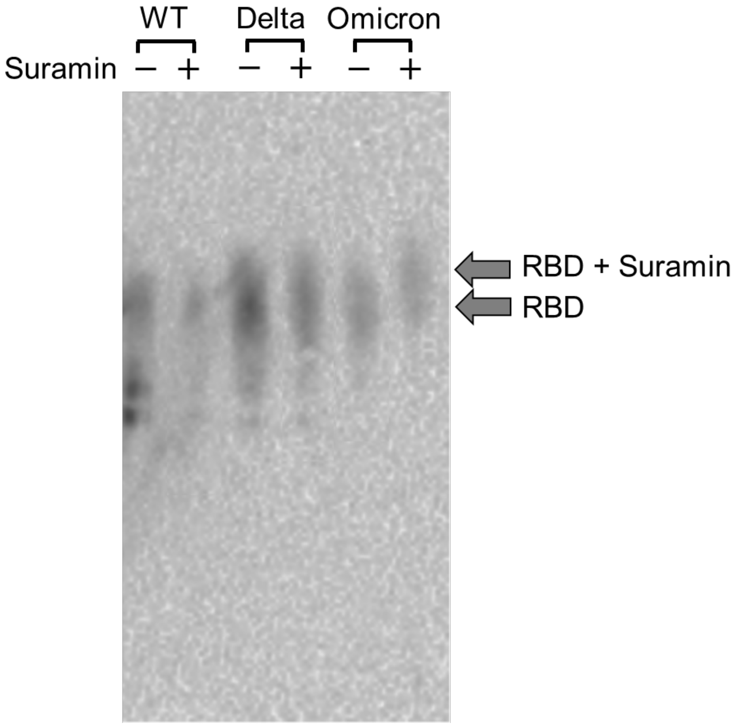

Supplementary Figure 1. Native PAGE and Western blot analysis of RBD variant and suramin-RBD variant complexes. Suramin-Omicron RBD complexes showed slightly increased size when compared to Omicron RBD alone.

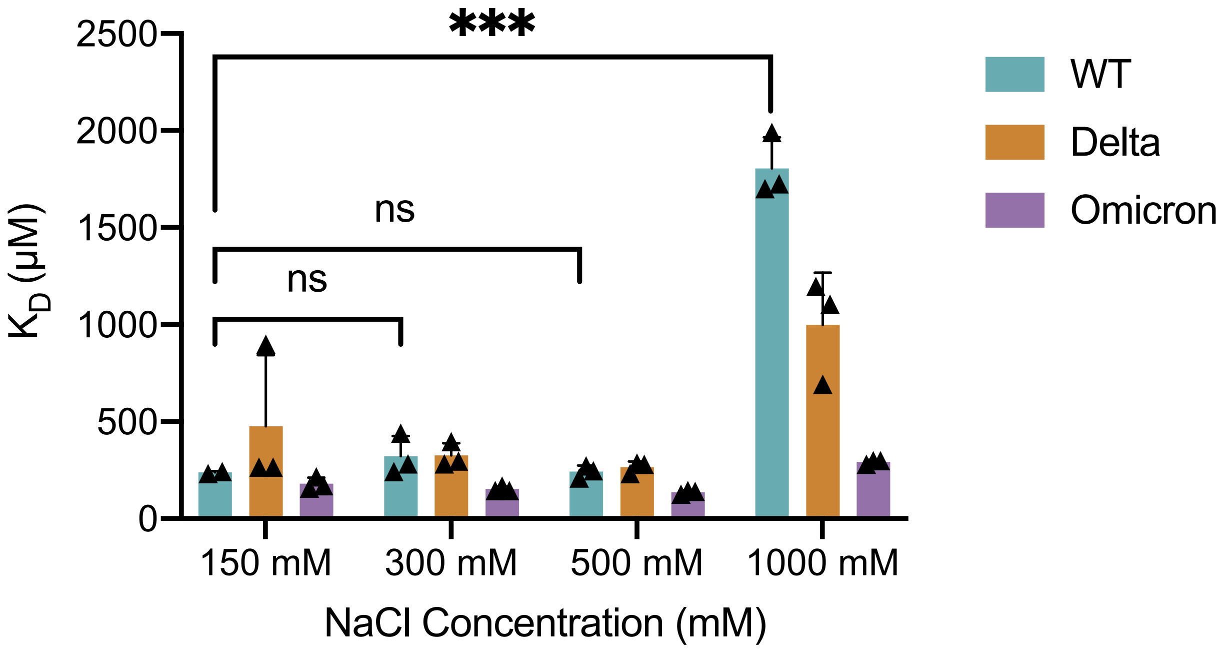

Supplementary Figure 2. Effect of salt (NaCl) concentration on the binding affinity K_D (μM) of suramin-SARS-CoV S-protein RBD variant interactions, based on triplicated SPR analysis. Error bars show mean ± sd. One statistical outlier (one data point for WT 150 mM NaCl) was removed.

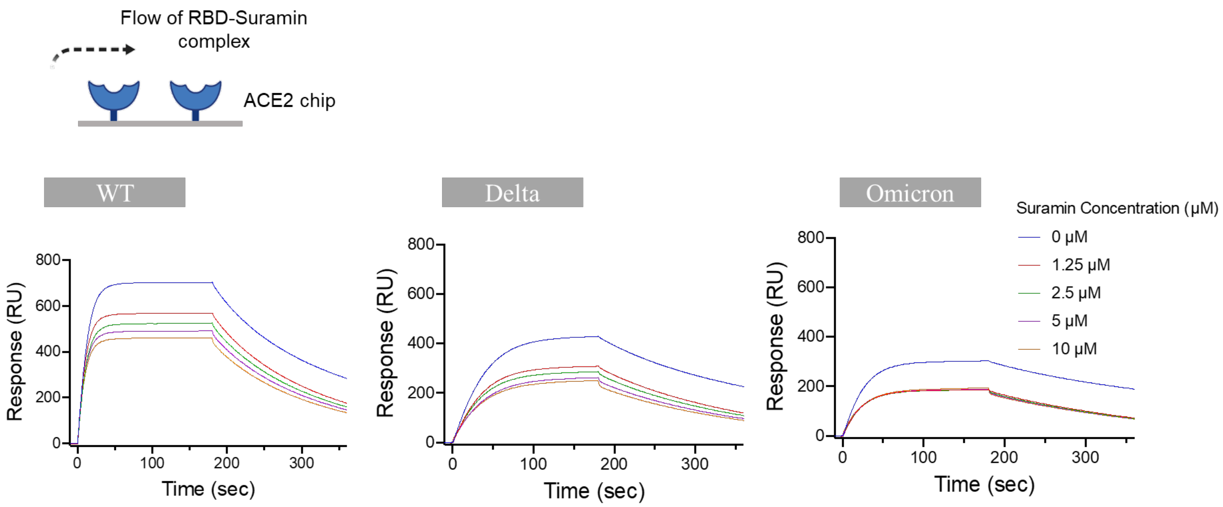

Supplementary Figure 3. Binding response of solution competition assay of suramin-RBD complex binding to ACE2 chip. Resulting sensorgram of binding at 250 nM of RBD with various concentrations of suramin is shown. For these experiments n = 3 replicates.

a

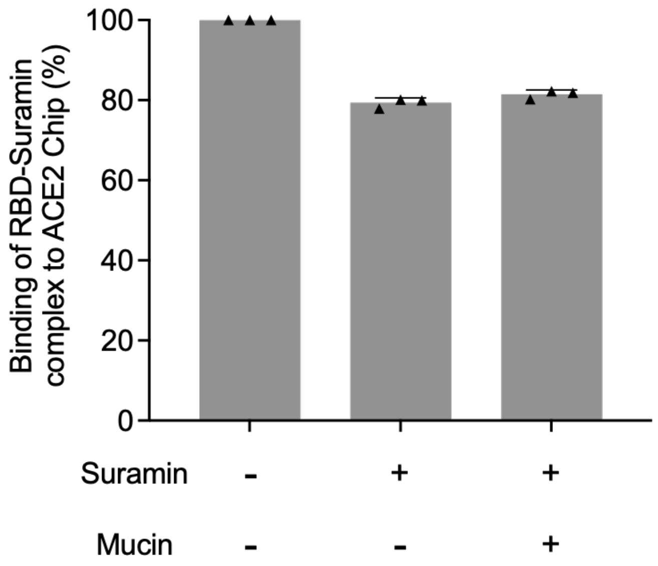

b

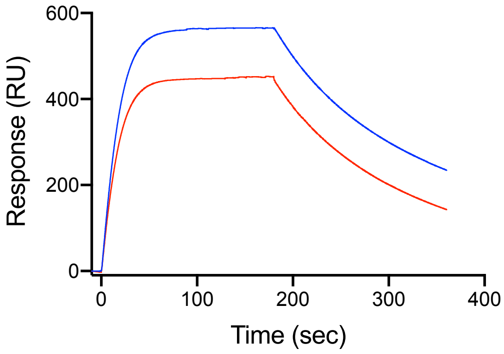

c

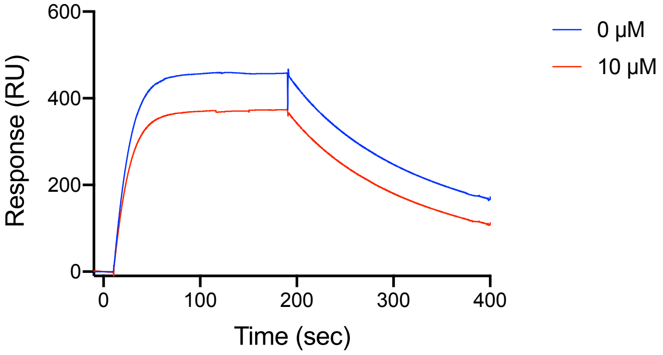

Supplementary Figure 4. (**a**) Binding response of solution competition assay of suramin-RBD complex binding to ACE2 chip in the presence of mucin. Sensorgrams for the binding of RBD-Suramin (250 nM RBD +/- 10 μM of suramin) complex to ACE2 chip in (**b**) HBS-EP+ buffer and (**c**) HBS-EP+ buffer with 0.5% (w/v) mucin. Error bars show mean ± sd.

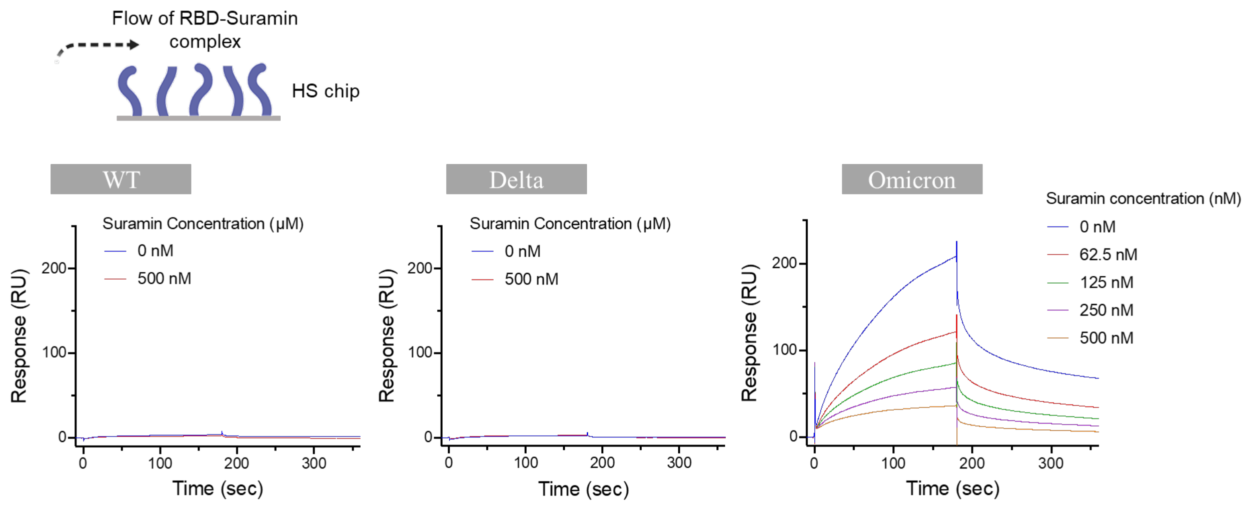

Supplementary Figure 5. Binding response of solution competition assay of suramin-RBD complex binding to HS chip. Resulting sensorgrams of binding at 250 nM of RBD with various concentrations of suramin is shown. For these experiments n = 3 biological replicates.

a.

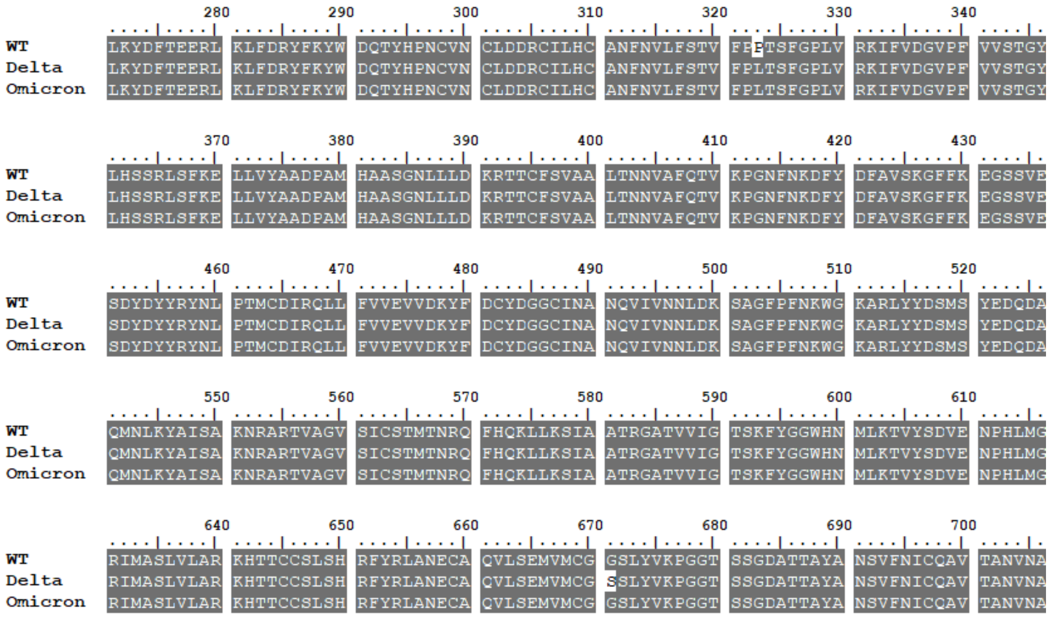

b

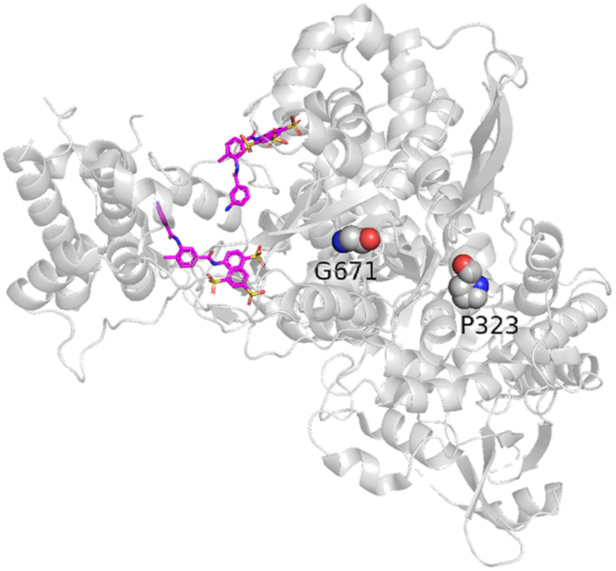

Supplementary Figure 6. (a) Blosum62 derived multiple sequence alignment (MSA) of SARS-CoV-2 RdRP variants. From MSA we observed that both the P323L and G671S mutations were found in Delta variant (B.1.617.2) and a P323L mutation was found in Omicron variant (B.1.1.529). (b) Molecular model of the SARS-CoV-2 RNA polymerase (PDB ID: 7D4F) with suramin bound. Here we observe the two binding site for suramin that are used to block binding to the RNA template and the other to restrict RNA primers. We can also observe on this structure that P323, and G671, the sites of point mutation in the Delta and Omicron variants are distal to the active site of the enzyme where suramin binds[1].

Supplementary References

1. Yin, W. *et al.* Structural basis for inhibition of the SARS-CoV-2 RNA polymerase by suramin. *Nat Struct Mol Biol* **28**, 319–325 (2021).
