# Supplementary material for: Suramin binds and inhibits infection of SARS-CoV-2 through both spike protein-heparan sulfate and ACE2 receptor interactions
Source: Commun Biol. 2023 Apr 8;6:387. doi: 10.1038/s42003-023-04789-z (PMC10082822; doi:10.1038/s42003-023-04789-z)
Supplement: Supplementary file 2 — Description of Additional Supplementary Files [file 42003_2023_4789_MOESM2_ESM.pdf]

Description of Additional Supplementary Files

File name: Supplementary Data
Description: The source data for figures in the paper.
